# Prognostic Implications of Heterogeneity in Intra-tumoral Immune Composition for Recurrence in Early Stage Lung Cancer

**DOI:** 10.3389/fimmu.2018.02298

**Published:** 2018-10-15

**Authors:** Jyothi Thyagabhavan Mony, Matthew J. Schuchert

**Affiliations:** Department of Cardiothoracic Surgery, University of Pittsburgh, School of Medicine, Pittsburgh, PA, United States

**Keywords:** lung adenocarcinoma, recurrence, CIBERSORT, CCL20, XCL1, plasma cells, Tregs, macrophages

## Abstract

**Background:** Studies in the past have identified selected immune cells that associate with different clinical outcomes in non-small cell lung cancer (NSCLC). Considering the fact that immune responses are heterogenous and that the clinical outcome could be influenced by the interplay of various immune cell types, it is imperative to evaluate multiple intra-tumoral immune cell types in the same set of patients.

**Objective:** To evaluate the individual and combined effects of diverse intra-tumoral immune cell types on recurrence after complete surgical resection in early stage lung adenocarcinoma.

**Methods:** We obtained NCBI GEO datasets for lung adenocarcinoma, the most prevalent histological subtype of NSCLC and re-analyzed the gene expression data of 292 patients with early stage cancer (IA/IB). CIBERSORT was used to resolve 22 immune cell types from the tumor transcriptomes. Survival analysis was carried out to assess the effect of immune cell types and genes associated with recurrence.

**Results:** Out of the 22 cell types, a high proportion of Tregs and monocyte-macrophages in the tumors were associated with significantly increased probability of recurrence. Conversely, increased proportion of non-Treg CD4+ T cells and plasma cells were associated with a lower probability of recurrence. The higher expression of *CCL20* (which can direct the migration of cells of B cell lineage), *XCL1* (associated with prototypical Th1 responses) and the immunoglobulin chains *IGHV4.34* and *IGLV6.57* were associated with a significantly lower probability of recurrence. Importantly, the intra-tumoral immune phenotype comprising these four cell types varied among patients and differentially associated with recurrence depending on net levels of positive and negative prognostic factors. Despite a high level of intra-tumoral plasma cells, a concomitant high level of monocyte-macrophages reduced the freedom from recurrence from ~80 to ~50% at 80 months (*p* < 0.05). Furthermore, stratification of the patients on the basis of a score estimated from the levels of four cell types enabled the identification of patients with significantly increased probability of recurrence (~50%) after surgery.

**Significance:** Our analysis suggests that concomitant levels of macrophages and plasma cells, in addition to the T regs and non-TregCD4+ T cells in tumors can identify patients with early stage lung cancer at greater risk of recurrence.

## Introduction

Lung cancer is a common malignancy and is the leading cause of cancer-related death in the United States and world-wide (1). NSCLC is the most prevalent subtype of lung cancer and its histological subtype lung adenocarcinoma constitutes over 50% of the cases of lung cancer diagnosed annually. Clinical decisions regarding treatment and cancer prognosis are primarily based on tumor stage as delineated by primary tumor size (T), degree of lymph node involvement (N) and detection of metastasis (M) (2). Surgical resection is the standard of care for patients with early stage NSCLC (3). Although surgery improves survival, lung cancer can recur in 20–25% of patients with clinical stage I disease (4). It is estimated that recurrence kills up to one-third of the lung cancer patients undergoing surgery (4, 5). Thus, recurrence poses a significant challenge to the treatment of lung cancer.

The immune system plays an intricate role in cancers. Immune responses involve highly coordinated interactions between numerous specialized cell types that can differentially influence the clinical outcome in NSCLC (6). The presence of tumor-infiltrating lymphocytes (TILs) are suggested to be associated with favorable prognosis in NSCLC (7–9). Several studies in the past have investigated the individual prognostic impact of selected immune cell types mostly on overall survival in patients with distinct histological subtypes of NSCLC in different stages (6). Furthermore, differences have been noted in the association of various immune cell types with the clinical outcomes in these studies suggesting a complex immunobiology of NSCLC. We hypothesized that the specific immune profiles comprising multiple cell types within resected tumors can efficiently predict recurrence in patients with early stage lung cancer undergoing surgery. Therefore, we carried out a detailed estimation of multiple immune cell types to identify patterns in immune composition of tumors that are associated with recurrence. We used CIBERSORT deconvolution software to infer the relative proportions of 22 distinct leukocyte cell types in the tumors from microarray gene expression data of early stage lung adenocarcinoma patients. Past studies have identified positive prognostic impact of plasma cells in overall survival (10, 11). Our analysis indicates that concomitant presence of macrophages has a negative impact on prognosis of recurrence and the estimates of plasma cell together with macrophages identifies patients at greater risk of recurrence. Furthermore, the estimates of Tregs, macrophages, non-Treg CD4+ T cells, and plasma cell, when considered together, can identify early stage lung adenocarcinoma patients at significantly greater risk of recurrence.

## Methods

### Datasets

A comprehensive search of the literature and Gene Expression Omnibus (GEO) database was carried out to identify microarray datasets with information on recurrence in patients with early stage lung adenocarcinoma. Microarray data from early stage lung adenocarcinoma patients (*n* = 293) from GEO datasets GSE68465, GSE37745 and GSE50081 were used in this study (12–14). Gene expression data from patients that received neo-adjuvant chemotherapy or radiotherapy were excluded from the analysis to avoid confounding factors. The study was restricted to datasets generated on Affymetrix human genome platforms that are compatible with the default reference leukocyte gene signature (LM22 matrix) in CIBERSORT (15).

### Re-analysis of microarray data

Raw microarray data (.CEL files) downloaded from GEO was normalized with MAS5 algorithm (affy package version 1.54.0, of Bioconductor version 3.5; in R 3.4.0), using custom chip definition files (CDF, 19.0.0) from Molecular and Behavioral Neuroscience Institute, University of Michigan was used for probeset summarization (16–18). Quality of arrays was evaluated using GNUSE function from fRMA package and a cutoff of 1.25 was used to filter bad arrays (19). One sample (GSM1672285) was excluded at this cutoff (Supplementary Excel). The datasets were quantile normalized and merged after adjusting for batch effects using “Insilicomerging” function (inSilicoDb package, Bioconductor version: 2.12, R 3.2.3) (20). Batch adjusted data was subsequently analyzed using CIBERSORT (1000 permutations) to resolve the immune composition.

### Gene expression analysis

Genes with low variability in more than 20 samples were excluded using “genefilter” function in “Genefilter” package (1.58.1). 3964 genes were retained for further analysis. Welch *T*-test was used to identify genes differentially expressed in tumors of recurrent and non-recurrent lung adenocarcinoma patients at threshold of unadjusted (nominal) *p*-value < 0.05. Reactome FIViz plug-in of the cytoscape biological network analysis platform was used to identify modules of highly-interacting groups of genes in lung adenocarcinoma (21, 22). The inflation parameter was set at 2.5 to identify modules with correlation >0.30. Cox proportional hazards (COXPH) regression was carried out using “coxfilter” function in “Genefilter” package (1.58.1) to assess the association of genes with recurrence outcome. A ranked gene list was generated from a comparison of recurrent and non-recurrent cases by Gene Set Enrichment analysis (GSEA) using immune gene sets from ImmuneSigDB (23, 24).

### Survival analysis

Pearson/Spearman correlation and ANOVA analysis (Dunn's multiple comparisons test) of immune cell proportions in tumors were performed on Graphpad PRISM (7.0c). Proportions of leukocytes from CIBERSORT and expression values of genes consistently identified by multiple methods were discretized into high and low categories based on mean values for each parameter using “quant.cut” function in R for Kaplan-Meier survival analysis (survival package, Bioconductor 3.5, R 3.4.0). Out of 292 samples meeting the microarray GNUSE cutoff, 12 were excluded due to lack of information on lack of follow-up information (Supplementary Excel). The p values are for the log rank test.

## Results

### Immune composition differs in recurrent and non-recurrent lung adenocarcinoma

Tumor stage positively correlated with recurrence (*r* = 0.15, *p* = 0.012). As expected, immune cells correlated differently with tumor stage and recurrence status (Table 1). The leukocyte RNA fraction estimated from neutrophils (*r* = 0.30, *p* < 0.0001) correlated significantly with tumor stage but not recurrence. Leukocyte RNA fraction from Treg (*r* = 0.19, *p* = 0.0019), M0-macrophages (*r* = 0.17, *p* = 0.0047) and M2-macrophages (*r* = 0.13, *p* = 0.036) correlated significantly with recurrence. Plasma cells (*r* = −0.16, *p* = 0.0069) inversely correlated with recurrence status.

**Table 1:** Correlation of leukocytes with recurrence and tumor stage.

**Correlation**	**Recurrence**	**Recurrence (*p*-value)**	**Tumor stage**	**Tumor stage (*p*-value)**
Tumor Stage	0.15	0.012		
Recurrence			0.15	0.012
B cells naive	0.00	0.946	0.07	0.262
B cells memory	0.10	0.108	−0.10	0.108
Total B cells	0.14	0.022	−0.05	0.368
Plasma cells	−0.16	0.007	−0.05	0.425
T cells CD8	0.00	0.967	0.13	0.030
T cells CD4 naive	−0.02	0.762	−0.05	0.376
T cells CD4 memory resting	−0.04	0.494	−0.14	0.022
T cells CD4 memory activated	−0.07	0.267	0.24	0.0001
T cells follicular helper	0.06	0.322	0.02	0.694
T regs	0.19	0.002	−0.01	0.927
Total T cells	−0.04	0.458	0.11	0.077
Tregs/NonTreg	0.19	0.001	−0.02	0.780
Total nonTreg CD4	−0.10	0.109	0.01	0.829
T cells gamma delta	−0.06	0.347	−0.01	0.824
NK cells resting	0.01	0.838	0.14	0.019
NK cells activated	0.06	0.311	0.05	0.452
Total NK	0.03	0.643	0.14	0.016
Monocytes	0.06	0.331	−0.09	0.116
M0-macrophages	0.17	0.005	0.03	0.614
M1-macrophage	0.00	0.987	0.09	0.141
M2-macrophages	0.13	0.036	0.02	0.737
Monocyte-macrophage system	0.19	0.001	0.01	0.831
Dendritic cells resting	0.09	0.154	−0.12	0.042
Dendritic cells activated	−0.02	0.733	−0.03	0.620
Dendritic cells	0.05	0.377	−0.11	0.057
Mast cells resting	0.00	0.964	−0.16	0.007
Mast cells activated	0.04	0.530	0.12	0.051
Eosinophils	−0.06	0.338	−0.15	0.010
Eosinophil-mast cells	0.06	0.345	−0.09	0.132
Neutrophils	0.02	0.724	0.30	0.0000005

*The percentage of leukocyte RNA contributed by various immune cell types estimated from tumor transcriptome data using CIBERSORT was used to evaluate correlation of leukocyte types with recurrence and tumor stage. (Full correlation matrix, Supplementary Excel)*.

**Figure 1:**
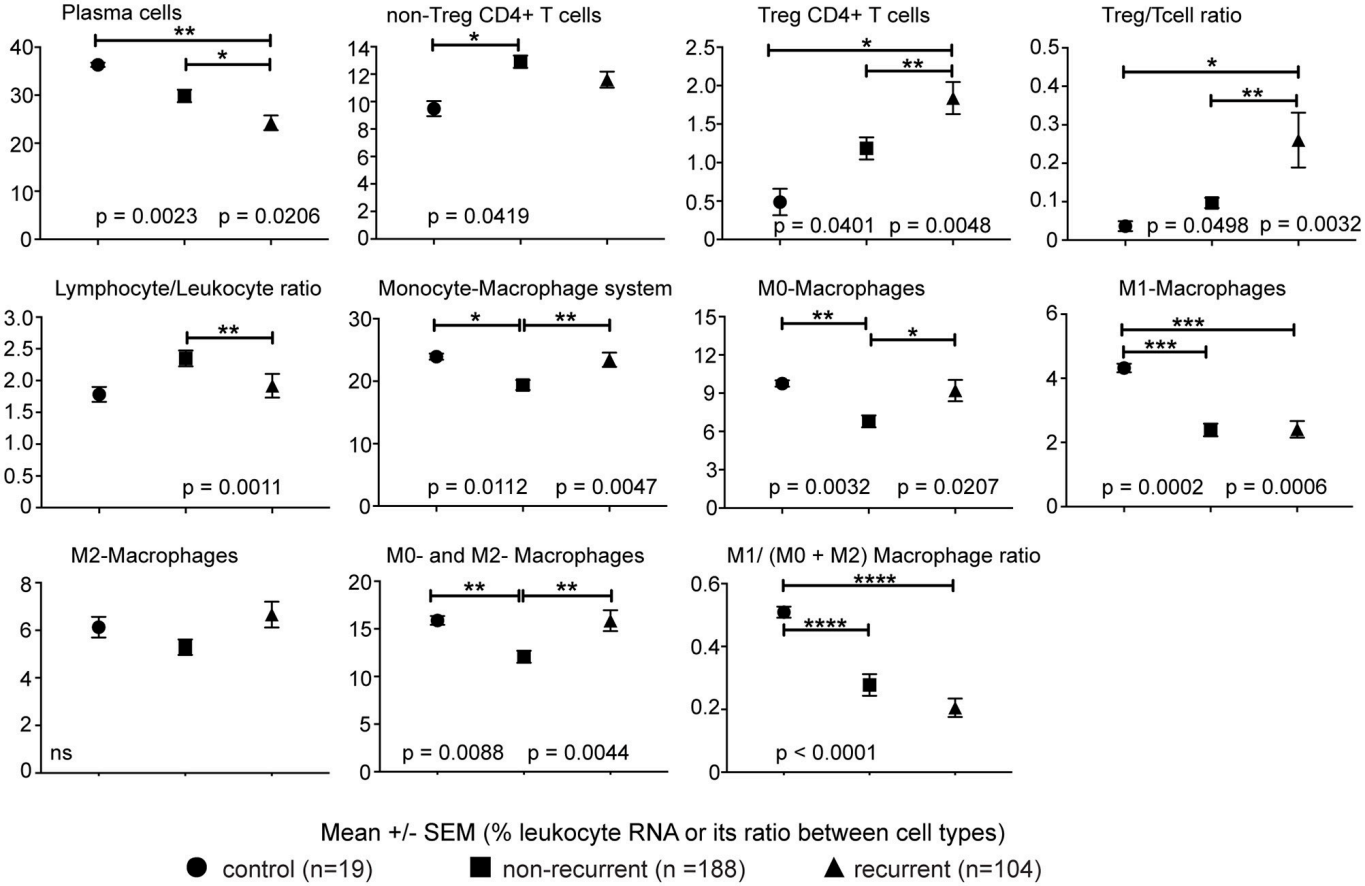
Immune cell types correlating with recurrence. The percentage of leukocyte RNA contributed by various immune cell types as estimated from tumor transcriptome data using CIBERSORT. Ratio of imputed percentages for Treg to rest of CD4+ T cells and lymphocytes to non-lymphocytic leukocytes was further used to confirm the differences in Treg and lymphocytes noted between recurrent and non-recurrent lung adenocarcinoma. Significant differences are denoted as ^*^p < 0.05, ^**^p < 0.01, ^***^p < 0.001 and ^****^p < 0.0001.

Significant differences were noted in the proportions of 22 immune cell types between tumors of patients with recurrent lung adenocarcinoma, non-recurrent lung adenocarcinoma and control lung samples (Figure 1). Plasma cells, cells of monocyte-macrophage lineage and T cells together constituted the majority of immune cell types contributing leukocyte RNA in tumors. A comparison of lymphocyte populations revealed significantly lower plasma cells (*p* = 0.0206) and increased Tregs (*p* = 0.0048) in recurrent lung adenocarcinoma. Consistent with the increased Treg percentages detected, Treg/T cell ratio was significantly higher (*p* = 0.0032) in tumors of patients with recurrent lung adenocarcinoma. Interestingly, increased non-Treg CD4+ T cell population was concurrently detected in non-recurrent category suggesting that dampening of effector CD4+ T cell responses by Tregs could play a role in recurrence. Consistent with this possibility, Tregs (*r* = −0.24, *p* < 0.0001) inversely correlated with activated CD4+ T memory (activeCD4+ Tmem) subset. In general, lymphocyte presence among leukocytes was significantly greater in tumors of patients with non-recurrent compared to recurrent lung adenocarcinoma (*p* = 0.0011) (Figure 1). In summary, recurrence is associated with increased Treg and reduced plasma cells in lung adenocarcinoma tumors.

In addition to changes in lymphocytes, we detected a significantly lower (*p* = 0.0047) proportion of cells of the monocyte-macrophage lineage in lung adenocarcinoma tumors of patients with non-recurrent disease. Although levels of monocytes and effector M1 and M2 macrophage subtypes were similar in tumors of non-recurrent and recurrent lung adenocarcinoma patients, a significantly lower proportion of immature M0-macrophage subtype (non-recurrent vs. control, *p* = 0.0032; non-recurrent vs. recurrent, *p* = 0.0207) was detected in non-recurrent lung adenocarcinoma (Figure 1). Consistent with a regulatory role for cells of monocytic lineage, plasma cells negatively correlated with monocytes, M0- and alternatively-activated M2-macrophages (*r* = −0.37, *p* < 0.0001; *r* = −0.15, *p* = 0.0098; *r* = −0.31, *p* < 0.0001, respectively). Consistent with its inflammatory role, the classically activated M1-macrophages correlated with active CD4+ Tmem and CD8+ T cells (*r* = 0.39, *p* < 0.0001; *r* = 0.43, *p* < 0.0001, respectively). Furthermore, M0-macrophages positively correlated with Treg (*r* = 0.30, *p* < 0.0001) and M1-macrophages inversely correlated with Tregs (*r* = −0.24, *p* < 0.0001) in tumors. The data suggests that monocyte-macrophage cells may play an important role in immune responses mediated by plasma and T cells. Furthermore, these differences in leukocyte composition of tumors suggests a possible role for differential leukocyte migration and/or retention in tumors.

### Non-lymphocytic leukocytes associated with greater probability of recurrence

**Figure 2:**
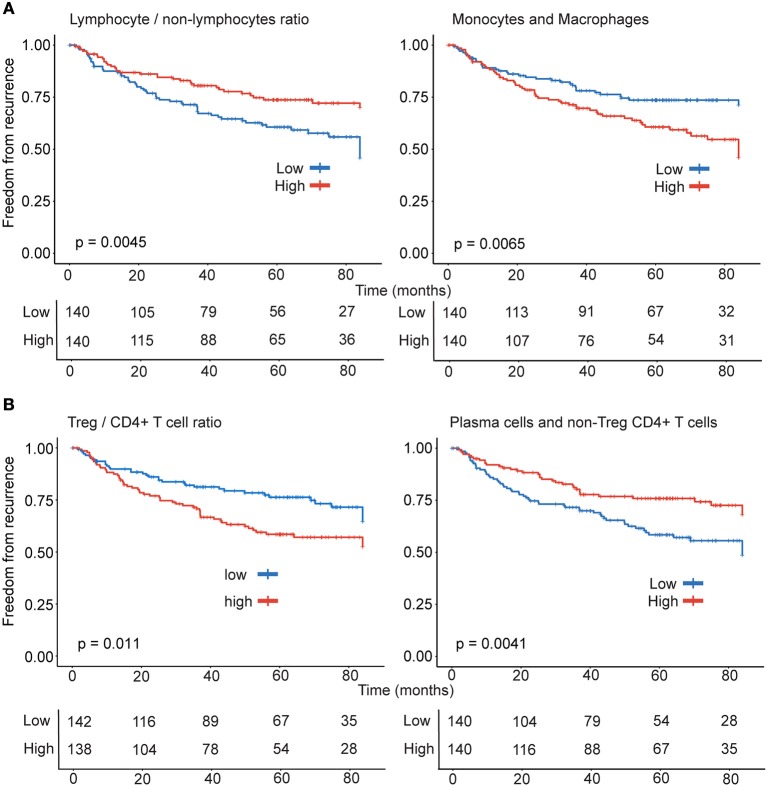
Differential effect of lymphocytes and monocytic lineage on recurrence. Patients (*n* = 280) were stratified into high (*n* = 140) and low (*n* = 140) categories based on the **(A)** ratio of lymphocyte and non-lymphocytic leukocytes and monocyte-macrophage subsets **(B)** ratio of Treg and non-Treg CD4+ T cells and the combination of heterogeneous immune effectors including non-Treg CD4+ T cells and plasma cells for Kaplan-Meier survival analysis.

Tumor stage is a predictor of prognosis and stage-Ib (*p* = 0.0062) was significantly associated with increased probability of recurrence in early stage lung adenocarcinoma. Survival analysis revealed that high levels of leukocytes of non-lymphocytic lineages (*p* = 0.0051) in tumors was significantly associated with greater probability of recurrence. In concordance with this observation, higher lymphocyte/non-lymphocyte ratio associated with lower recurrence (*p* = 0.0045) (Figure 2A). Consistent with the correlation of leukocytes of monocytic lineage with recurrence, the probability of recurrence was significantly greater when tumors had increased presence of monocytes and macrophages (*p* = 0.0065; Figure 2A).

### Lymphocytes differentially associated with probability of recurrence

Regulatory CD4+ T cells play an important role in dampening responses driven by effector T cells. The effector activated CD4+ T memory (active CD4+ Tmem) in non-Treg CD4+T cell population, correlated with M1-macrophages (*r* = 0.39, *p* < 0.0001) and cytotoxic lymphocytes CD8+ T cells (*r* = 0.41, *p* < 0.0001) and NK cells (*r* = 0.25, *p* < 0.0001). In concordance with the literature on clinical effect of Tregs in NSCLC prognosis, tumors with high levels of Tregs (*p* = 0.0073), Treg/T cells (*p* = 0.011) and Treg/non-Treg CD4+ T cell ratio (*p* = 0.011) (Figure 2B) had significantly greater probability of recurrence. Among the rest of the lymphocytes, only plasma cells (*r* = −0.16, *p* = 0.007, Table 1) inversely correlated with recurrence. Thus, suggesting that effector responses involving CD4+ T cells and plasma cells could favorably shape clinical outcome in patients with lung adenocarcinoma. Categorizing patients on the basis of plasma cells and non-Treg CD4+ T cell levels in tumors identified that higher proportion of both plasma cells and non-Treg CD4+ T cells (*p* = 0.0041) in tumors were significantly associated with reduced probability of recurrence (Figure 2B).

### Chemokines and immunoglobulins associated with lower probability of recurrence

**Figure 3:**
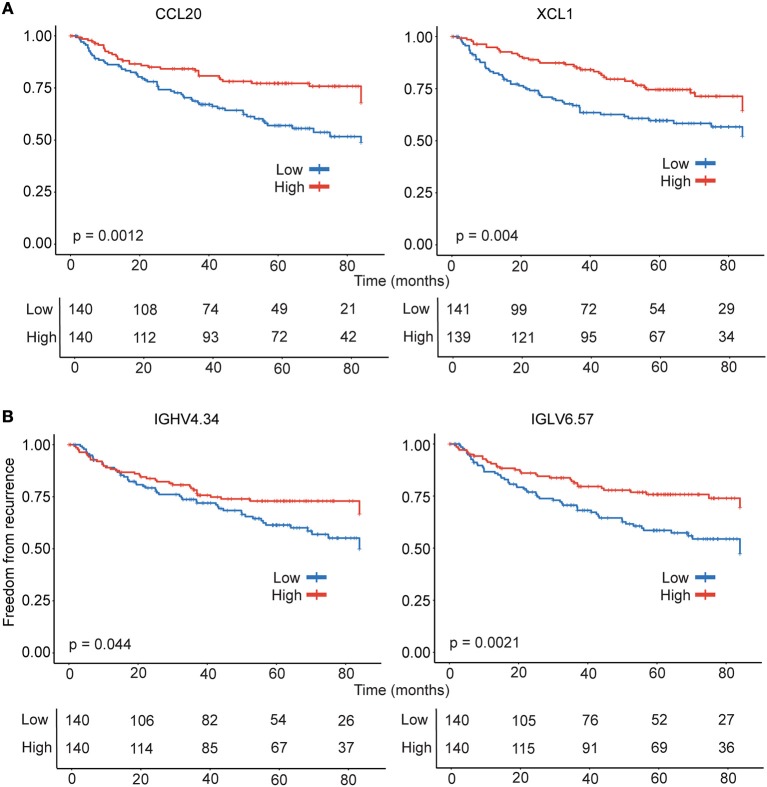
Kaplan- Meier estimates of time to recurrence for genes tightly linked to freedom from ecurrence. Survival analysis was performed for chemokine **(A)** and immunoglobulin **(B)** genes consistently identified by multiple approaches. The gene expression data was used to stratify the patients (*n* = 280) were stratified into high (*n* = 140) and low (*n* = 140) categories for Kaplan-Meier survival analysis.

The analysis of the tumor transcriptome using CIBERSORT revealed significant differences in intra-tumoral leukocyte composition suggesting differential leukocyte migration and/or retention in tumors. Furthermore, a significant reduction in plasma cell percentages suggested weaker antibody responses in recurrent disease. Therefore, we hypothesized that genes involved in leukocyte migration and function as well as antibody responses could have a clinical significance in recurrence. To identify candidate genes for survival analysis, we re-analyzed the microarray data in Reactome FIViz in Cytoscape and identified 19 genes from the modules in lung adenocarcinoma that were either identified as significantly associated with recurrence in COXPH analysis and/or were also detected as differently expressed in patient groups at nominal *p* < 0.05 cut-off. Furthermore, out of the 19 genes, (3/7) immunoglobulins, (2/3) chemokines and (2/2) MHC-II were detected amongst the lowest ranked 5% of genes in the ranked gene list generated by GSEA to identify genes associated with recurrence, suggesting an important role for robust antibody responses, leukocyte migration and lymphocyte function in tumors of early stage lung adenocarcinoma. Importantly, 4 genes were significantly associated with recurrence in Kaplan-Meier analysis. The higher expression of chemokines, *CCL20* (*p* = 0.0012) that can direct migration of cells of B cell lineage, and *XCL1* (*p* = 0.004) associated with prototypical Th1 immune response, were associated with significantly lower probability of recurrence (Figure 3A). Furthermore, in concordance with the reduced intra-tumoral plasma cells in the recurrent category estimated using CIBERSORT, decreased expression of the immunoglobulin genes *IGHV4.34* (*p* = 0.044) and *IGLV6.57* (*p* = 0.0021) were associated with significantly higher probability of recurrence (Figure 3B). Importantly, while CCL20 is also part of LM22 matrix, the genes *XCL1, IGHV4.34*, and *IGLV6.57* associated with greater freedom from recurrence are identified independent of CIBERSORT analysis and suggests a robust T cell-mediated and mature B cell/Plasma cell-mediated immune response in the tumors of patients with non-recurrent disease.

### Net estimates of the leukocytes predict greater probability of recurrence

**Figure 4:**
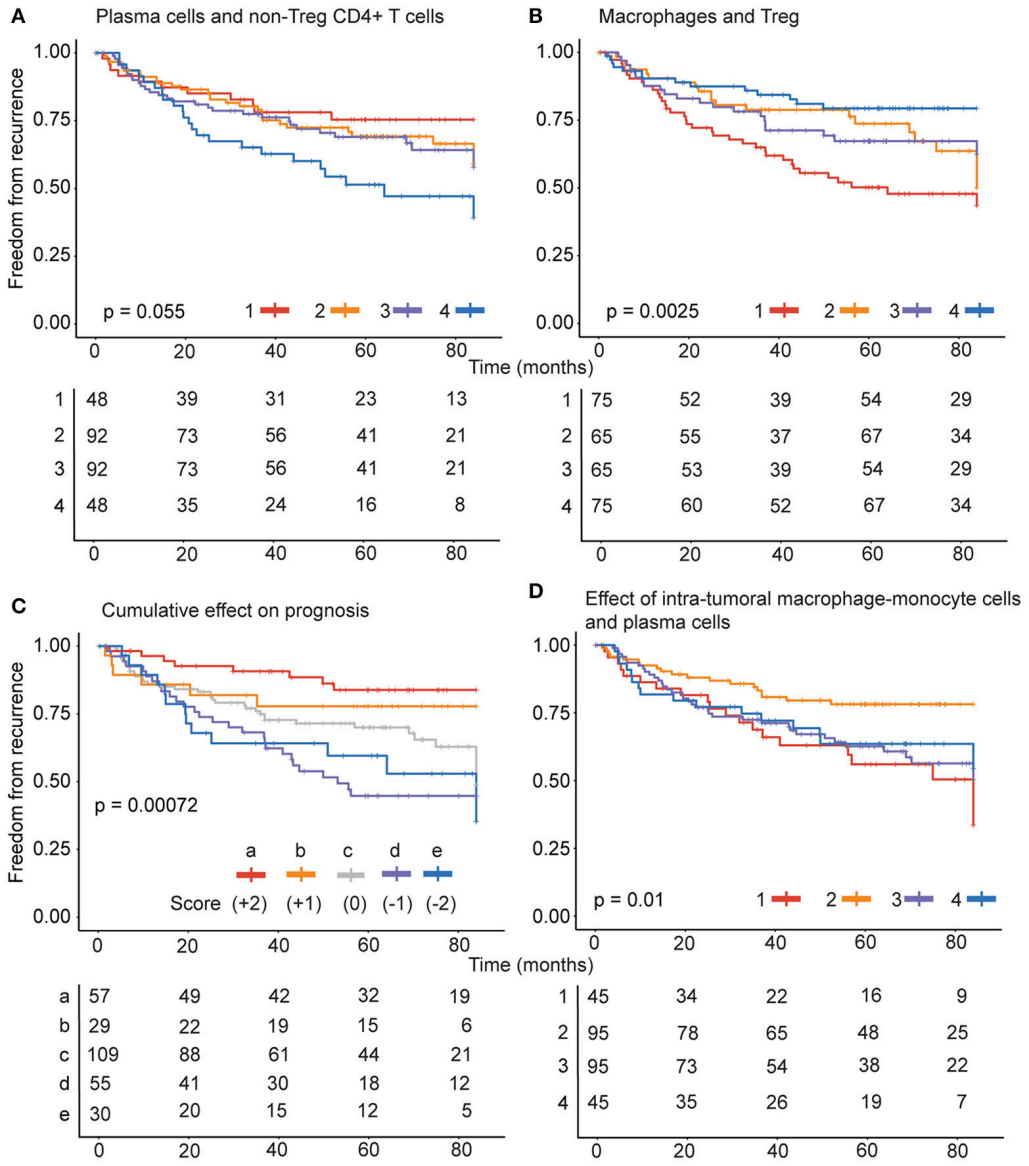
Combined effect of prognostic variables on recurrence. **(A)** For negative prognostic factors, patients were classified into (1) Treg (high), monocyte-macrophage (high), (2) Treg (high), monocyte-macrophage (low) (3) Treg (low), monocyte-macrophage (high) and (4) Treg (low), monocyte-macrophage (low) categories. **(B)** For positive prognostic factors, the patients were classified into (1) non-Treg CD4+ T cell (high), plasma cell (high), (2) non-Treg CD4+ T cell (high), plasma cell (low) (3) non-Treg CD4+ T cell (low), monocyte-macrophage (high) and (4) non-Treg CD4+ T cell (low), plasma cell (low) categories. **(C)** The patients were stratified into 5 groups (a–e) based on the score estimated from the levels of each of the negative and positive prognostic cell types and **(D)** specifically on the basis of monocyte-macrophage and plasma cells, into (1) plasma cell (high), monocyte-macrophage (high), (2) plasma cell (high), monocyte-macrophage (low) (3) plasma cell (low), monocyte-macrophage (high) and (4) plasma cell (low), monocyte-macrophage (low) categories.

Intra-tumoral immune composition of patients varied with respect to the four cell types and therefore, we sought to identify intra-tumoral immune-phenotype in patients with greater risk. We analyzed the probabilities of recurrence in 280 patients categorized into high and low groups based on distinct prognostic combinations of immune cells identified. Patients were classified into (1) non-Treg CD4+ T cell (high), plasma cell (high), (2) non-Treg CD4+ T cell (high), plasma cell (low) (3) non-Treg CD4+ T cell (low), plasma cell (high) and (4) non-Treg CD4+ T cell (low), plasma cell (low) categories for analyzing the significance of positive prognostic factors (Figure 4A). Patients were also classified into (1) Treg (high), monocyte-macrophage (high), (2) Treg (high), monocyte-macrophage (low) (3) Treg (low), monocyte-macrophage (high) and (4) Treg (low), monocyte-macrophage (low) categories based on negative prognostic factors (Figure 4B). The combination of negative prognostic factors identified a subset of patients with an immune phenotype comprising high levels of Treg and monocyte-macrophage with increased probability of recurrence (~50%) after surgery. Also, patients with low levels of non-Treg CD4+ T cells and plasma cells also had an increased probability of recurrence (~50%).

Importantly, variations in the positive and negative prognostic factors differently influenced the freedom from recurrence in early stage lung adenocarcinoma (Figure 4C). The collective impact of the four immune cells was evaluated by stratifying the patients into 5 groups (a-e) based on levels of each of the negative and positive prognostic cell types. For each cell type a patient was scored as high (1) or low (0) as above. The score for plasma cells and non-Treg CD4+ T cell were added to obtain a positive prognostic score on a 0–2 scale, where 0 represented low and 2 represented high levels of both cell types and 1 represented high levels for one cell type. Likewise, score for Tregs and monocyte-macrophage cells were added to obtain a negative prognostic score on a 0–2 scale. The positive prognostic score was subtracted from the negative prognostic score for each patient to determine the category for stratification.

The analysis revealed that the presence of four cell types in the tumors play a complex role in recurrence outcome. As expected high levels of both plasma cells and non-Treg CD4+ T cells (score +2) contributed to greater freedom from recurrence. However, the presence of high levels of Tregs and/or monocyte-macrophage cells significantly dented the freedom from recurrence (scores 0, −1, and −2). The effect of negative prognostic cell types is most evident when patients are stratified on the basis of intra-tumoral monocyte-macrophage cells and plasma cells, into (1) plasma cell (high), monocyte-macrophage (high), (2) plasma cell (high), monocyte-macrophage (low) (3) plasma cell (low), monocyte-macrophage (high) and (4) plasma cell (low), monocyte-macrophage (low) categories. Importantly, despite high plasma cells that inversely correlated with recurrence, patients with concomitant high levels of monocyte-macrophage cells in the tumors had the poorest freedom from recurrence (Figure 4D, strata 1).

## Discussion

Immune responses are heterogeneous and can involve multiple cell types that can differentially influence clinical outcome. In this regard, differences have been noted among studies that have evaluated the individual prognostic effect of select few immune cell types on overall survival or recurrence in NSCLC as reviewed by Remark et al. (6). Furthermore, immune composition is different in adenocarcinoma and squamous cell carcinoma (SCC) subtypes of NSCLC suggesting a complex immunobiology of lung cancer (25). The goal of our study was to estimate diverse leukocyte types in the tumors and identify key immune cell types or their combinations that associate with recurrence of early stage lung adenocarcinoma following surgical resection. In order to study the immune composition of lung adenocarcinoma, we adopted a proven *in-silico* approach using CIBERSORT to resolve 22 immune cell types from tumor transcriptome (11, 15, 26). Among the immune cell types estimated, Tregs, macrophages, non-Treg CD4+ T cells, and plasma cells had significant association with recurrence outcome. However, intra-tumoral immune phenotype comprising these four cell types varied among patients and in its association with recurrence. Importantly, we identified a subset of early stage lung cancer patients with an “at risk” intra-tumoral immune phenotype and significantly increased probability of recurrence (~50%).

CD4+ T cells play an important role in shaping adaptive immune responses. Regulatory FOXP3+CD4+ T cells are a subset of CD4+ T helper cells that inhibit adaptive immune responses driven by various CD4+ and CD8+ T cell effectors (27). Tregs and its relative abundance among TILs are consistent predictors of recurrence in NSCLC (28–31). In concordance with the literature, our analysis of lung adenocarcinoma transcriptome with CIBERSORT showed that Tregs correlated with increased recurrence, and the probability of recurrence was significantly higher when immune composition of tumors was rich in Treg (high Treg/T cell ratio). Conversely, a low probability of recurrence was noted when Treg/T cell ratio was low and the levels of non-Treg CD4+ T cells were high in the tumors. Furthermore, Tregs inversely correlated with active CD4+ Tmem in lung adenocarcinoma.

Analysis of T cell response in tumors revealed that active CD4+ Tmem cells correlated with M1-macrophages, cytotoxic lymphocytes CD8+ T cells and NK cells associated with Th1 responses. While the Treg subset of CD4+ T cells had a significant negative prognostic effect, the rest of CD4+ T cell population (non-Treg CD4+ T cells) in the tumors had a favorable prognostic effect. Though, we could not estimate the different CD4+ T cell subsets including Th1, Th2, and Th17 due to the current limitation of CIBERSORT reference panel, our analysis of gene expression patterns revealed that the chemokine *XCL1* has a significant effect in regulating recurrence. *XCL1* is suggested to be associated with Th1-skewed immune responses and is highly expressed by Th1-differentiated CD4+ T cells, CD8+ T cells, and NK cells (32). Thus, our data suggests the possibility that Th1-type immune response plays an important role in regulating recurrence in early stage.

Typically, protective anti-tumor immune responses have been attributed to cytotoxic CD8+ T cells that can eliminate tumor cells (27). However, CD8+ T cells have a variable impact on clinical outcome in NSCLC, thus suggesting a complex role in NSCLC (31, 33–39). Mori et al. (33) did not detect a correlation between CD8+ T cells infiltrating the tumors and survival. Though, Wakabayashi et al. (34) found no correlation between CD8+ T cell infiltration and prognosis in NSCLC cases, higher number of CD8+ T cells in tumors correlated with poor 5y-survival rates, especially in lung adenocarcinoma. In contrast, Goc et al. (39), Kawai et al. (37). and Schalper et al. (38) found infiltrating CD8+ T cells to favorably associate with survival. While Al-Shibli et al. (36) identified both tumor-infiltrating CD4+ and CD8+ T cells as independent favorable prognosis markers, the Hiraoka et al. (35) study identified that a concomitant infiltration of tumors by CD8+ and CD4+ T cells, but not CD8+ T cells alone, was associated with better prognosis. This lack of concordance in literature suggest that CD8+ T cell functional status rather than numbers, associate with lung cancer. In line with this hypothesis, CD8+ T cells did not differ significantly between groups or correlate with recurrence. The lack of significant differences in CD8+ T cell estimates in recurrent and non-recurrent lung adenocarcinoma does not exclude the possibility of differences in functional state of CD8+ T cells. CD8+ T cell activation is suggested to result in XCL1 expression and subsequent recruitment of XCR1-expressing DCs that can further enhance the immune response by cross-presentation of antigens to CD8+ T cells (40, 41). We have identified that reduced XCL1 expression is associated with increased risk of recurrence in the patients. This suggests the possibility that robust natural cytotoxic lymphocyte response involving CD8+ T and/or NK cells is ineffective in lung adenocarcinoma patients with recurrent disease.

Humoral immune function is suggested to serve as prognostic marker in several solid tumors (10). The B/plasma cell signature, *IGKC* mRNA expression, and plasma cell levels inferred from transcriptome analysis were significantly associated with longer survival in NSCLC (10, 11). Our data from transcriptome analysis extends these findings and indicates an important role for antibody-mediated immune responses in recurrence of early stage lung adenocarcinoma in patients undergoing curative surgery. Importantly, our data brings forth the limitation of plasma cell estimates alone as a prognostic marker (Figure 4D). We have identified a subset of patients with high levels of plasma cells and concomitant high levels of monocytic cells with greater probability of recurrence that was among the worst for the patients studied. Thus, emphasizing the need to evaluate multiple cell types in prognosis of cancer recurrence. The significantly reduced levels of plasma cells in tumors of recurrent cases (Figure 1) suggests that migration of the cells of B cell lineage is hampered. The chemokine ligand *CCL20* and its receptor *CCR6* play an important role in recruiting circulating memory B cells to sites of inflammation (42). *CCR6* expression in B cell lineage is restricted to functionally mature cells capable of responding to antigen challenge (43). CCL20 was picked up by multiple gene expression analysis approaches and is also part of CIBERSORT LM22 matrix. This gene is identified as significantly differentially expressed in activated CD4+ T memory subset, activated dendritic cells and mast cells (15). The expression of CCL20 could represent robust CD4+ T cell response and efficient recruitment of functionally antibody-producing B cell types to the tumors. In this regard, we found that patients with increased probability of recurrence had significantly lower levels of *CCL20* expression in the tumors. Thus, our data suggests an important role for CCL20-CCR6 axis in contributing to humoral anti-tumor immune response in early stage lung cancer.

The heterogeneous tumor-infiltrating myeloid cell population comprised of cells of neutrophil and monocyte lineages can suppress lymphocyte function (44). In our study, the cells of the monocyte-macrophage lineage, in particular, the immature M0-macrophages and alternatively-activated M2-macrophages significantly correlated with recurrence. Consistent with an association with poor prognosis, the higher levels of monocyte-macrophage lineage were associated with a significantly higher probability of recurrence. Neutrophils are suggested to be a strong predictor of mortality in NSCLC patients (11). Kargl et al. (25) found that neutrophils and monocytes in NSCLC tumors enumerated using flow cytometry correlated with tumor stage in lung adenocarcinoma. Consistent with Kargl et al. (25), neutrophils correlated with tumor stage in our analysis. However, we have found that neutrophils neither correlated nor had significant association with recurrence in early stage lung adenocarcinoma.

Interestingly, T regulatory cells, M0- and M2-macrophages were strongly associated with poor clinical outcome in a CIBERSORT analysis of breast cancer transcriptome data in public domain that included the TCGA database (26). Our analysis (Figures 4C,D) identifies a complex effect of negative prognostic cell types (Tregs and monocyte-macrophage cells) and positive prognostic cell types (plasma cells and non-Treg CD4+ T cells) on clinical outcome and suggests a conserved cellular mechanism that hinders tumor clearance by lymphocyte-driven immune responses that in turn could influence recurrence in early stage lung cancer patients.

Our approach permitted a comprehensive analysis of the intra-tumoral immune composition in early stage lung cancer and identifies distinct intra tumoral immune profiles comprising both positive and negative prognostic immune cell types that can influence recurrence. The localization of immune cells identified could not be evaluated further since this is a retrospective study based on tumor transcriptome data in the public domain. Gene expression does not always correlate with protein levels in the cells and differential transcriptional activity among cell types can potentially inflate the estimation of transcriptionally active plasma cells or deflate the less active neutrophils (25). This confounding effect is minimized by using the output for identifying the differences in each of the immune cell types among the tumor samples. We have also used ratios of cell types wherever possible. Furthermore, as CIBERSORT uses only a limited set of genes (547) to estimate the 22 immune cell types, we hypothesized that independent analysis of the tumor transcriptome would identify more genes associated with the relevant cell types. Consistent with the CIBERSORT output, we identified genes involved in T cell- and antibody-mediated immune responses and leukocyte migration that associated with greater freedom from recurrence. Importantly, our findings are consistent with the past studies in NSCLC that have evaluated the prognostic significance of select few cell types independently and indicates the robustness of the approach.

The TNM staging system guides the decisions regarding treatment and prognosis in lung cancer. However, differential prognosis has been noted in patients with same TNM stage, thus highlighting the need to identify factors that can enhance the prognostic functionality of TNM staging system. The immunoscore projects evaluating immune markers in cancers are currently focused on T cells (45). Our results prioritize the immune cell types constituting a prognostic phenotype for validation in prospective studies in early stage lung cancer and has implications for the development of immunoscore in lung cancer. Importantly, our study identifies a subset of early stage lung cancer patients with increased probability of recurrence based on “at risk” immune phenotype characterized by intra-tumoral composition of increased Treg and macrophage populations, as well as decreased non-Treg CD4+ T cells and plasma cells. This “at risk” phenotype can complement the standard TNM tumor stage in early stage NSCLC by identifying patients with increased probability of recurrence following curative surgery.

## Author contributions

JM and MS were responsible for the conception and design of the study. The analysis was performed by JM. JM and MS were involved in interpretation of the data. The manuscript was drafted by JM. JM and MS have critically revised the manuscript for the intellectual content and gave final approval for its submission.

### Conflict of interest statement

The authors declare that the research was conducted in the absence of any commercial or financial relationships that could be construed as a potential conflict of interest.
